# Suicidal behaviors and attention deficit hyperactivity disorder (ADHD): a cross-sectional study among Chinese medical college students

**DOI:** 10.1186/s12888-021-03247-6

**Published:** 2021-05-18

**Authors:** Yanmei Shen, Bella Siu Man Chan, Chunxiang Huang, Xilong Cui, Jianbo Liu, Jianping Lu, Marguerite Patel, Christopher D. Verrico, Xuerong Luo, Xiang Yang Zhang

**Affiliations:** 1 China National Clinical Research Center for Mental Disorders, and Department of Psychiatry, The Second Xiangya Hospital of Central South University, Changsha, 410011 Hunan China; 2 The Department of Educational and Counselling Psychology, and Special Education, The University of British Columbia, Vancouver, Canada; 3 Department of Child and Adolescent Psychiatry of Shenzhen Kangning Hospital, Shenzhen Mental Health Center, Shenzhen, China; 4 Department of Psychiatry and Behavioral Sciences, Baylor College of Medicine, Houston, TX USA; 5 Department of Psychiatry and Behavioral Sciences, The University of Texas Health Science Center at Houston, UT Houston Medical School, Houston, TX 77054 USA; 6 Institute of Psychology, Chinese Academy of Sciences, Beijing, China

**Keywords:** ADHD, Suicidal behavior, Odds ratio, Anxiety, Depression, Epidemiology

## Abstract

**Backgrounds:**

Associations between attention deficit hyperactivity disorder (ADHD) subtypes and suicidal behaviors remains unclear. The current study explored the prevalence of suicidal behaviors, and its association with ADHD among Chinese medical students.

**Methods:**

Five thousand six hundred ninety-three medical college students participated. Symptoms of suicidal behaviors, ADHD, anxiety, depression, tobacco and alcohol use were assessed using online questionnaires.

**Results:**

The prevalence of lifetime suicidal ideation, suicide plans, and suicide attempts among medical college students were 27.5, 7.9 and 14.8% respectively. Participants with ADHD predominantly inattentive type (ADHD-I) had more than fivefold increased odds of suicidal behaviors, the adjusted odds ratios (ORs) of ADHD-I and ADHD combined type (ADHD-C) remained significant after controlling for confounding factors.

**Conclusions:**

ADHD is associated with high risk of suicidal behaviors. ADHD-I and ADHD-C were strongly associated with suicidal behaviors independent of comorbidities. The finding suggests the importance of addressing ADHD symptoms in suicide prevention.

## Background

For people 15–29 years old around the world, suicide is the second leading cause of death [1]. 1.4% of all human deaths are caused by suicide and each year 800,000 people commit suicide globally [2]. Despite increasing prevention efforts, current trends predict that by 2020, nearly 1.53 million people will die by suicide [3]. More people die from suicide than from war, AIDS, car accidents, or homicide [4]. In China, suicide is one of the top five causes of death for its nearly 1.3 billion citizens [5]. Suicide prevention is a serious public health challenge and further research of suicidal behaviors is crucial as such behavior is a warning and opportunity to prevent fatal attempts [6].

Attention-deficit hyperactivity disorder (ADHD) is a neurodevelopmental disorder which begins in childhood. Children are usually diagnosed in their school-age years, after presenting with inattention, hyperactivity, and impulsivity symptoms causing functional impairment [7]. ADHD prevalence estimates are 5.29–7.1% in childhood and adolescence, and 1.2–7.3% in adulthood [8–10]. ADHD symptoms can persist into adulthood and have lasting effect, with studies suggesting an association between ADHD and increased risk of cognitive impairments, antisocial behavior, depression, substance abuse, and loss of inhibition [11]. Recent studies have shown that individuals with ADHD are at an increased risk of engaging in suicidal behaviors [12–14], they suggested that ADHD symptoms were associated with the three kinds of suicidal behaviors [15]. with odds ratios ranging from 3.84 ~ 6.52 [12–14, 16]. While some studies have suggested that ADHD may increase suicide risk due to its comorbidities, there has been less research on the direct association between suicidal behaviors and ADHD independent of its comorbidities [17, 18]. One study has found that individuals with ADHD, regardless of comorbidities, are at a greater risk of attempting and completing suicide [19].

Data have consistently shown that medical students report high levels of suicidal ideation, plans, and attempts relative to the rest of the population. This is true both in China and across the globe [20–23]. However, there has been little research examining the predictors of suicidal behaviors of medical students, especially in China. Since medical students go on to become future clinicians, their early mental health is particularly important given its longitudinal influence on patient care and service quality [24–26]. Our recent study reported that the prevalence of ADHD in medical students was 3.5%, and ADHD was significantly associated with suicidal ideation, suicide plans, or suicide attempts, indicating a close association between ADHD and suicidal behaviors [27]. As a result, gaining a better understanding of the associations between suicidal behaviors and ADHD is crucial for providing further direction for suicide prevention.

Although there is a already a meta analysis revealed the association between ADHD and suicidal behaviors [28], few studies examined the association between ADHD subtype and suicidal behavior. This study was the first of its kind to explore the relationship between ADHD subtypes and suicidal behavior in Chinese medical students. The research goals were as follows: 1) to determine the prevalence of suicidal behaviors among medical students; 2) to examine which ADHD subtypes associate with which suicidal behaviors, and the strength of these associations and 3) how these associations are impacted by demographic factors and other mental health comorbidities. Understanding both general and specific associations between ADHD and suicidal behaviors is of great importance in assisting efforts to stratify suicide risk and improve prevention efforts.

## Methods

### Participants

Research approval was granted by the Ethics Committee of the Second Xiangya Hospital at Central South University, China. From a convenience sampling, a total of 5822 undergraduates were selected from three medical colleges (5577 from YiYang Medical College, 80 from Hunan University of Chinese Medicine and 165 from Changsha Health Vocational College). Both Yiyang Medical College and Changsha Health Vocational College employ the 3 year undergraduate system, students attending this medical program had relatively poorer academic achievements. Hunan University, on the other hand, offers a 4 year comprehensive medical program, thus participants tend to have higher academic achievements compare to the former two colleges.

Five thousand eight hundred twenty-two college students were screened and were provided voluntary consent forms to sign between January and March, 2018. Of these, 116 declined to participate and thirteen participants were excluded because of unqualified questionnaires. A total of 5693 participants completed the study.

### Measures

Before beginning the study, college instructors and counselors were trained in data collection and participant instruction, after which they instructed participants on how to complete the questionnaires below, either by computer online or on Wechat, a common social media app.

#### Demographic information

We utilized a self-report scale for age, nationality, gender, community, family type, socioeconomic status, education level of parents, and tobacco and alcohol use.

#### ADHD

We utilized two measures, 1) the Wender Utah Rating Scale (WURS), brief Chinese version, a 15-item questionnaire to assess a childhood history of ADHD symptoms [29]; and 2) the World Health Organization (WHO) Adult ADHD Self-Report Scale v 1.1 Symptom Checklist (ASRS) an 18 question battery to assess current ADHD symptoms in adulthood [30]. Both WURS and ASRS have been shown to have satisfactory psychometric properties within China [30, 31]. A positive result was indicated by a total score of 30 or higher in WURS [29], and 17 or higher in any ASRS subscale (include inattention subscale and hyperactivity/impulsivity subscale) [32]. Participants who met the criteria on both scales were considered to have a diagnosis of ADHD for the purposes of our study. Participants with ADHD were further divided into three subtypes: participants with scores higher than 17 on both inattention and hyperactivity/impulsivity subscales were considered to be ADHD combined presentation (ADHD-C), those with scores higher than 17 in inattention subscale were considered to be ADHD predominantly inattentive presentation (ADHD-I), and those with scores higher than 17 in hyperactivity/impulsivity subscale were considered to be ADHD predominantly hyperactive/impulsive presentation (ADHD-H).

#### Suicidal behaviors

Lifetime suicidal behaviors were defined as the presence of suicidal ideations, plans, and/or past attempts [33]. Suicidal ideation was defined as either passive wishes for death or active thoughts about committing suicide. Suicide plans included any plans or preparations for a suicide attempt. Finally, suicide attempts were defined as any potentially self-injurious actions the individual takes against themselves, with at least some degree of an intent to die [33]. Participants were asked three questions regarding suicidal ideation, plans and attempts: 1) “Have you ever had thoughts of committing suicide?”; 2) Have you ever made a suicide plan?”; and 3) Have you ever tried committing suicide?”. If participants responded positive to these questions, they will continue to ask questions that inquire about the method, frequency and further relevant details on the attempts of the suicide. These questions were adapted from the Columbia-Suicide Severity Rating Scale [34].

Based on participants’ responses to questions concerned about suicidal behaviors, participants were divided into 4 main groups. Participants without any kind of suicidal behaviors were divided into control group, subjects exhibiting the specific type of suicidal behaviors were further divided into three subgroups with suicidal ideations, plans and attempts. Since overlapping occurred among three subtypes of suicidal behavior, those subjects with two or three subtypes of suicidal behavior were included in two or three subgroups.

#### Anxiety and depression

Symptoms of anxiety and depression during the prior week were evaluated using Zung’s Self-Rating Anxiety Scale (SAS) and Self-Rating Depression Scale (SDS), with each scale including 20 self-report items on a one-to-four score range [35, 36]. A subject was considered to have clinically-relevant anxiety with a score of 50 or higher on SAS, and depression with a score of 53 or higher on SDS, based on normative Chinese diagnostic criteria [37]. SAS and SDS have shown satisfactory psychometric properties in China, and both have been used widely for members of the Chinese population [38, 39].

### Data analysis

Subjects were divided into four groups based on their report of 1) no suicidal behaviors, 2) suicidal ideation, 3) suicide plans, and 4) suicide attempts. Clinical and demographic variables between groups were compared using chi-squared tests. Crosstabs were used to calculate the crude odds ratio (OR) between variables and suicidal behaviors, and binary logistic regression method was used to calculate the adjusted OR with correction for confounding variables. Statistical analysis was conducted using SPSS 21, with two-tailed *p* values of 0.05.

## Results

The mean age of the study sample (*N* = 5693) was 18.40 ± 1.49 years, distributed as following: 18.51 years (SD = 1.47) for controls, 18.11 years (SD = 1.50) for suicidal ideation, 17.99 years (SD = 1.43) for suicide plans, and 17.96 years (SD = 1.46) for suicide attempts. Amongst all participants, 1565 (27.5%) reported lifetime suicidal ideation, 450 (7.9%) reported lifetime suicide plans, and 841 (14.8%) reported lifetime suicide attempts. Table 1 summarizes the characteristics of the study sample for participants without suicidal behaviors (control) versus those with suicidal ideation (SI), suicide plans (SP), and suicide attempts (SA). Participants with suicidal behavior were more likely to be female, living in urban communities, be of Han nationality, have a health condition, have a family history of mental disorders, have a poor relationship with parents, use tobacco and alcohol, and have clinical anxiety or depression.

**Table 1:** Demographic characteristics of control group and participants with suicidal behaviors

Variables	Controls *N* = 4046	SI *N* = 1565	*P*	OR	SP *N* = 450	*P*	OR	SA *N* = 841	*P*	OR
**Males/females**	483/3563	119/1446	< 0.001	1.647 (1.336–2.032)	37/413	0.019	1.513 (1.067–2.146)	71/770	0.004	1.470 (1.132–1.909)
**District: Rural/Urban**	2976/1070	1078/487	< 0.001	1.256 (1.106–1.428)	288/162	< 0.001	1.564 (1.275–1.920)	568/273	< 0.001	1.337 (1.139–1.569)
**Nationality: Others/Han**	487/3559	152/1413	0.014	1.272 (1.050–1.542)	34/416	0.005	1.674 (1.166–2.405)	72/769	0.004	1.461 (1.127–1.894)
**Good relationship with mother**	3960 (97.9%)	1471 (94.0%)	< 0.001	0.34 (0.252–0.458)	407 (90.4%)	< 0.001	0.206 (0.141–0.301)	780 (92.7%)	< 0.001	0.278 (0.198–0.389)
**Good relationship with father**	3906 (96.5%)	1418 (90.6%)	< 0.001	0.346 (0.272–0.439)	389 (86.4%)	< 0.001	0.229 (0.166–0.314)	747 (88.8%)	< 0.001	0.285 (0.217–0.374)
**Physical disorder history**	196 (4.8%)	168 (10.7%)	< 0.001	2.362 (1.905–2.929)	52 (11.6%)	< 0.001	2.566 (1.859–3.544)	100 (11.9%)	< 0.001	2.651 (2.058–3.415)
**Family history of mental disorder**	60 (1.5%)	48 (3.1%)	< 0.001	2.102 (1.432–3.086)	23 (5.1%)	< 0.001	3.578 (2.190–5.846)	33 (3.9%)	< 0.001	2.713 (1.762–4.177)
**Smoking**	146 (3.6%)	90 (5.8%)	< 0.001	1.63 (1.245–2.134)	44 (9.8%)	< 0.001	2.895 (2.035–4.117)	69 (8.2%)	< 0.001	2.388 (1.775–3.212)
**Drinking**	549 (13.6%)	351 (22.4%)	< 0.001	1.842 (1.587–2.138)	128 (28.4%)	< 0.001	2.532 (2.025–3.167)	229 (27.2%)	< 0.001	2.383 (1.998–2.844)
**Anxiety**	601 (14.9%)	594 (38.0%)	< 0.001	3.507 (3.067–4.009)	225 (50.0%)	< 0.001	5.732 (4.674–7.030)	388 (46.1%)	< 0.001	4.991 (4.180–5.767)
**Depression**	1070 (26.4%)	829 (53.0%)	< 0.001	3.133 (2.775–3.537)	271 (60.2%)	< 0.001	4.211 (3.443–5.150)	497 (59.1%)	< 0.001	4.018 (3.444–4.688)
**ADHD**	59 (1.5%)	135 (8.6%)	< 0.001^a^	6.38 (4.671–8.714)	59 (13.1%)	< 0.001^a^	10.197 (7.005–14.844)	97 (11.5%)	< 0.001^a^	8.810 (6.315–12.291)
ADHD-I	26	71	< 0.001^b^		28	< 0.001^b^		50	< 0.001^b^	
ADHD-H	4	9			4			7		
ADHD-C	29	55			27			40		

Note: *SI* Suicidal ideation, *SP* Suicide plans, *SA* Suicide attempts, *ADHD-I* Attention deficit hyperactivity disorder predominantly inattentive type, *ADHD-H* Attention deficit hyperactivity disorder predominantly hyperactive-impulsive type, *ADHD-C* Attention deficit hyperactivity disorder combined type; ^a^is the comparison between healthy controls and those three suicidal behaviors, ^b^is a comparison among the three types of ADHD

Participants with ADHD were more than six times likely to have suicidal ideation compared to those without ADHD, with an OR (95%CI) of 6.38(4.67–8.71) for suicidal ideation, 10.20(7.01–14.84) for suicide plans, and 8.81(6.32–12.29) for suicide attempts (see Table 1). The crude OR of suicidal ideation for ADHD-I, ADHD-H, ADHD-C was 7.61(4.84–11.98), 6.27(1.93–20.40), and 5.29(3.36–8.33), respectively, for suicide plans the crude OR was 10.98(6.38–18.92), 10.20(2.54–40.93), and 9.49(5.56–16.20), respectively, and 10.31(6.38–16.66), 9.38(2.74–32.12), and 7.39(4.55–12.00) for suicide attempts. The ORs remained significant after adjustment for demographic confounders and comorbidities including gender, rural-urban classification, nationality, parental relationship with the participants, comorbid health conditions, family psychiatric history, smoking, drinking alcohol, anxiety and depression. The adjusted OR for suicidal ideation was 4.38(2.72–7.06), 3.33(0.99–11.22) and 2.37(1.45–3.88) for ADHD-I, ADHD-H and ADHD-C respectively, after adjusting for anxiety, depression, and substance use. In addition, the adjusted ORs of ADHD-I and ADHD-C for suicide plans and attempts remained significant (see Table 2).

**Table 2:** Associations between suicidal behaviors and subtypes of ADHD, with and without adjustment for demographic confounders and comorbidities

Variable	ADHD-I OR (95% CI)	*P*	ADHD-H OR (95% CI)	*P*	ADHD-C OR (95% CI)	*P*
**Suicidal ideation**						
Crude	7.614 (4.839–11.980)	0.000^***^	6.273 (1.929–20.402)	0.010^*^	5.288 (3.359–8.325)	0.000^***^
Adjusted for demographics and comorbidities	4.381 (2.721–7.055)	0.000^***^	3.332 (0.989–11.222)	0.052	2.374 (1.453–3.876)	0.001^**^
**Suicide plans**						
Crude	10.981 (6.375–18.916)	0.000^***^	10.197 (2.540–40.931)	0.011^*^	9.494 (5.564–16.199)	0.000^***^
Adjusted for demographics and comorbidities	5.648 (3.125–10.208)	0.000^***^	3.865 (0.861–17.347)	0.078	2.805 (1.506–5.225)	0.001^**^
**Suicide attempts**						
Crude	10.306 (6.375–16.660)	0.000^***^	9.378 (2.738–32.115)	0.002^**^	7.392 (4.554–11.998)	0.000^***^
Adjusted for demographics and comorbidities	5.153 (3.082–8.617)	0.000^***^	3.704 (1.020–13.458)	0.047^*^	2.879 (1.668–4.969)	0.000^***^

Note: ^*^p < .05; ^**^p < .01; ^***^p < .001; *ADHD-I* Attention deficit hyperactivity disorder predominantly inattentive type, *ADHD-H* Attention deficit hyperactivity disorder predominantly hyperactive-impulsive type, *ADHD-C* Attention deficit hyperactivity disorder combined type

## Discussion

The high prevalence of suicidal behaviors in this large sample study of medical college students implies a serious mental health problem and public health concern. We also found a strong association between ADHD and suicidal behaviors; participants with ADHD are at six to ten times the risk of having suicidal behaviors as compared to those without. ADHD-I and ADHD-C remain independent risk factors for suicidal behaviors even after adjusting for confounding factors, including demographics and comorbid psychiatric diagnoses.

### Prevalence

Around the world, college students are a specific group reporting high levels of suicidal ideation, plans, and attempts [23, 40, 41]. Prevalence of suicidal ideation – the most common of suicidal behaviors – was 27.5% in this study. This was a much higher prevalence rate than the pooled prevalence reported in an international meta-analysis of students from 15 countries (*n* = 21,002) by Rotenstein et al. [42] . It was also significantly higher than the pooled prevalence of 10.72% derived from 41 160,339 Chinese college students [43], although individual studies within that population have reported a higher range of prevalence between 6 to 39.2% [44]. The possible explanation for higher rates of suicidal ideation specifically in medical college students is due to fierce competition for medical school admissions. Thus, the medical college students have more stress and higher rate of depression compared to the general population [45, 46] as well as the other college students [23, 42, 46]. Another possible explanation is that the resistance within the medical college students to help-seeking, since studying medicine is recognized to cause stress; however, the medical students reported prevailing perceptions of stigma associated with mental illness, including stress and help-seeking may be regarded as a form of weakness [47]. There is also known inter-study variation due to gender ratio, response rate, and sample size, as suggested by Li et al., that these factors explain almost half of the heterogeneity between studies [43]. Also, the use of different screening tools may influence this heterogeneity [43]. In addition, cultural origins may also contribute to the heterogeneity of prevalence. For example, a study found that Asian Americans are at a higher risk of suicidal behaviors and are less likely to seek help due to stigma of mental disorders. Meanwhile, Asian American parents put more emphasis on “saving face”, which prevent them from discussing their mental health issues with other people [48].

This study found a higher prevalence of suicide attempts than suicide plans, 14.8% over 7.9% respectively, in all college students. This reveals that nearly half of the participants who had made an attempt at suicide had done so without planning it, which is consistent with previous research finding that over 20% of suicide attempts were serious but non-fatal, and approximately 75% of attempts involved no preparations or plans beforehand [49]. One explanation for this high rate of suicide attempts may be related to the current educational system in China. The students’ academic achievement is mainly accentuated on obtaining knowledge through exam-oriented assessments, as a result there has been too little focus on the development and improvement of the overall psychological wellness in the individuals [50]. When facing upon unbearably stressful situations, the students with poor psychological quality, low pressure resistance and pessimistic emotions were unable to rationally self-cope, which made them lack the capacity to deal with frustration and tend to attempt suicide impulsively [50]. Another explanation may be related to the way on how we have assessed the participants’ suicidal behaviors. In this study, the participants were assessed whether they had made suicide plans and attempts by one simple question: “Have you ever made a suicide plan/attempts?” If the response to the question is yes, then other questions put forward on how they had tried to hurt themselves and what frequency of their attempts were. In addition, there may be a possibility that the participants did not understand fully the definition of the term “suicide plan”, which may have resulted in a lower rate of suicide planning than attempts. Future studies should include a detailed and clear definition of suicide plan to alleviate any ambiguity or confusions. Finally, impulsivity may be an important explanation of the difference between suicide plan and suicide attempt, as people with ADHD often tend to suffer from a lack of planning.

### Association between ADHD and suicidal behavior

This study found a significant association between ADHD and suicidal behaviors, consistent with prior research [19, 51, 52]. One explanation is that ADHD is frequently comorbid with other psychiatric disorders [53, 54], and both on their own as well as in conjunction with ADHD, psychiatric disorders are proven to carry a higher risk of poor clinical outcomes and increase risk of hospitalization and mortality [54, 55]. However, there is still no consensus as to whether increased risk of ADHD is only mediated through comorbidity with other psychiatric disorders, or if it represents an independent risk factor. While Jame et al. concluded in a review that ADHD increases the risk of suicidal behaviors solely through comorbid conditions [56], other research has shown that even after adjusting for comorbid disorders, ADHD carries an independent increased risk for suicidal behavior [16, 19, 51]. One explanation for such inconsistent conclusions may be that previous studies did not examine suicidal behaviors based on differentiated ADHD subtypes. It is possible that each ADHD subtype may have varying associations with different suicidal behaviors.

To explore this possibility, our study examined in closer detail the association of these subtypes with suicidal behaviors. We found that ADHD-H was strongly associated with suicidal behaviors but failed to remain significant after adjusting for comorbid disorders, implying that only through comorbid psychiatric disorders does hyperactivity/impulsivity increase the risk of suicidal behaviors. However, ADHD-I and ADHD-C were significantly associated with suicidal behaviors, which persisted even after adjusting for comorbid disorders and demographic characteristics. These results indicate that inattention may be a very important impairing factor, which is consistent with several previous studies [51]. However, this finding was inconsistent with some other research [56, 57]. Hinshaw et al. reported that suicide attempts were associated solely with hyperactive/impulsive symptoms; yet this difference may be due to a relatively smaller sample size (*N* = 140), population limitation (only females recruited), and investigation of suicide attempts only [57]. Future research should include larger sample sizes among broader demographic and cultural populations, and should include all forms of suicidal behaviors, in order to gain a more clear representation. Finally, although previous studies have addressed the importance of treating ADHD [58–60], they have not paid much attention to the high risk of suicidal behaviors in patients with ADHD, and future studies should pay more attention to the risk of suicidal behaviors.

This study had several limitations. First, suicidal behavior data was collected through self-reports of the participants themselves. Therefore, the reported prevalence rates are relatively high, which might have an influence on the validity of the results, future studies should utilize both questionnaire and diagnostic interview to assess the participants’ suicidal behaviors. Recall-bias should be taken into account when inpterprete the results of tthis study. Second, participants were determined to have ADHD, anxiety, or depression when their self-reported scores met criteria. While the scales used in this study have been shown to possess good psychometric properties, evidence does exist suggesting there may be bias in reporting symptoms [61, 62]. Third, some unknown confounders that may regulate the relationship between suicidal behaviors and ADHD were not included in this study such as the comorbidity of ODD, the students’ ADHD status: “childhood ADHD”, “persistent ADHD”, physical injuries [63], poisoning [64], etc. Future research should include these variables to better examine potential association mechanisms, and how the interplay of various comorbidities affect the risk of suicidal behavior. Fourth, the participants in this study were medical college students, therefore, the findings of this study could not be generalized to the whole populations. Fifth, the participants were primarily female and there are few persons in the subgroup ADHD-H which might generate a selection bias; future research should include larger sample size and more male participants to gain better representation of the population as a whole. Six, considering the small sample size of ADHD-H subtype, the data and statistical power to support the conclusions on ADHD subtypes might be insufficient. Future studies will benefit from incorporating larger sample size. Finally, suicidal behaviour was assessed by questionnaires, therefore, the reported prevalence rates are relatively high, which might have an influence on the validity of the results, future studies should utilize both questionnaire and diagnostic interview to assess the participants’ suicidal behaviors.

## Conclusions

This study shows a high prevalence of suicidal behaviors among medical college students, and that individuals with ADHD have a six-to-ten-time higher risk of suicidal behaviors. The findings of this study have important clinical implications. First, it is imperative to attend to the mental health of medical college students – a high-risk population – in order to prevent suicide. Second, the early diagnosis and treatment of ADHD may also serve in the role of suicide prevention. Healthcare professionals should keep both of these high risk groups in mind when treating patients.

## Data Availability

The data that support the findings of this study are available on request from the. corresponding author. The data are not publicly available due to privacy or ethical restrictions.

## Abbreviations

ADHD: Attention Deficit Hyperactivity Disorder
ADHD-I: Attention deficit hyperactivity disorder predominantly inattentive type
ADHD-H: Attention deficit hyperactivity disorder predominantly hyperactive-impulsive type
ADHD-C: Attention deficit hyperactivity disorder combined type
OR: Odds ratio
WHO: World Health Organization
WURS: Wender Utah Rating Scale
ASRS: Adult ADHD Self-Report Scale v 1.1 Symptom Checklist
SAS: Self-Rating Anxiety Scale
SDS: Self-Rating Depression Scale
SI: Suicidal ideation
SP: Suicide plans
SA: Suicide attempts
